# Supplementary material for: Highly ordered macroporous hydrogen-bonded organic frameworks based on small biocompatible molecules
Source: Nat Commun. 2025 Dec 8;17:2968. doi: 10.1038/s41467-025-67123-7 (PMC13035950; doi:10.1038/s41467-025-67123-7)
Supplement: Supplementary file 1 — Supplementary Information [file 41467_2025_67123_MOESM1_ESM.docx]

Supplementary Information

**Highly Ordered Macroporous Hydrogen-Bonded Organic Frameworks Based on Small Biocompatible Molecules**

Qiu-Xia Li, Wan-Zhen Cai, Xiao-Liang Ye, Yi Zeng, A. R. Mahammed Shaheer, Zai-Sheng Ye and Tian-Fu Liu*

**Contents**

1. Instruments 2

2. Molecular packing structure images 2

3. Powder X-ray diffraction (PXRD) patterns 3

4. N_2_ adsorption-desorption isotherms 3

5. Scanning electron microscopy (SEM) and transmission electron microscopy (TEM) 5

6. Solvent stability 8

7. Fourier transform infrared (FT-IR) spectra 10

8. Ultraviolet-Visible (UV-Vis) spectroscopy 10

9. Storage stability 14

10. Reusability 15

11. Cell culture conditions and fibrocyte differentiation assay 16

Supplementary References 18

**Experimental Section**

1. **Instruments**

Powder X-ray diffraction (PXRD) patterns were recorded on a Rikagu Miniflex 600 Benchtop equipped with Cu Κα radiation (λ= 0.154 nm). N_2_ adsorption-desorption isotherms and the Brunauer-Emmett-Teller (BET) surface area measurements were performed using a Micromeritics ASAP 2460 instrument. UV-Visible (UV-vis) absorption spectra were recorded on a Shimadzu UV-2550 spectrophotometer. Fourier transform infrared (FT-IR) spectra were collected using a VERTEX70 series FT-IR spectrometer. Scanning electron microscopy (SEM) images were obtained using a JSM6700-F scanning electron microscope. Transmission electron microscopy (TEM) studies were performed on a JEM-F200 TEM.

1. **Molecular packing structure images**

**
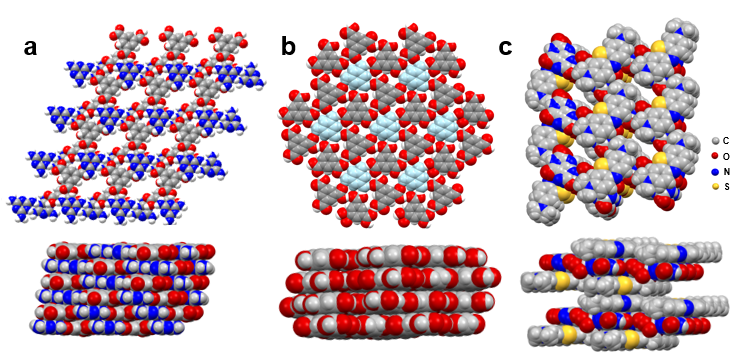
**

**Supplementary Figure 1** **|** **Structure of the samples.** Molecular packing structure images of (a) MA-TMA, (b) THB-TMA and (c) MBU co-crystal.

**Supplementary Table 1 |** **Toxicological information.** The toxicological information of the OM-HOFs’ building blocks.

| **Sample** | | **Oral: LD_50_ (mg/kg)** |  |
| --- | --- | --- | --- |
| MA | 4550 | | |
| TMA | 16000 | | |
| THB | 5200 | | |
| MB | 3500 | | |
| UA | 5040 | | |

1. **Powder X-ray diffraction (PXRD) patterns**

**
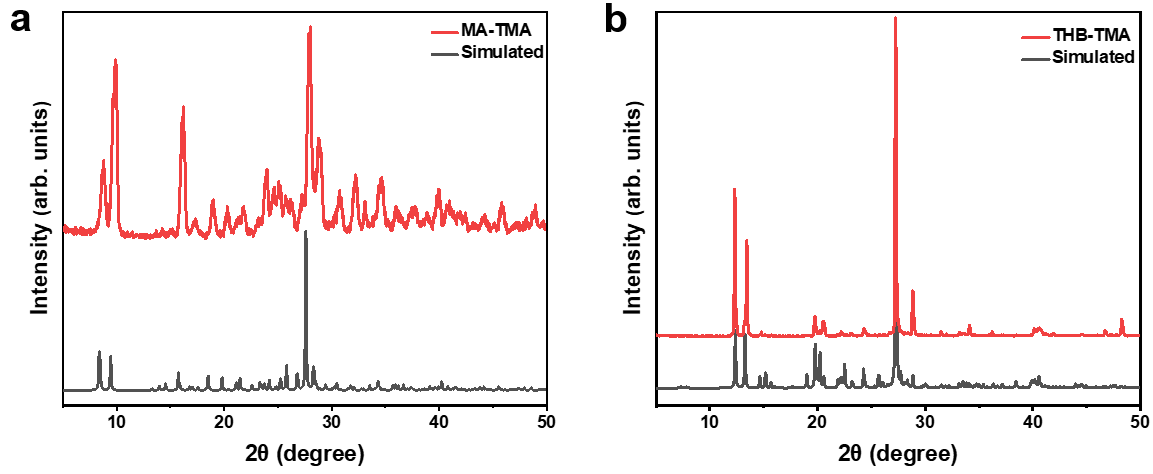
**

**Supplementary Figure 2 | Structure of the samples.** The PXRD patterns of (a) MA-TMA and (b) THB-TMA co-crystal.

**
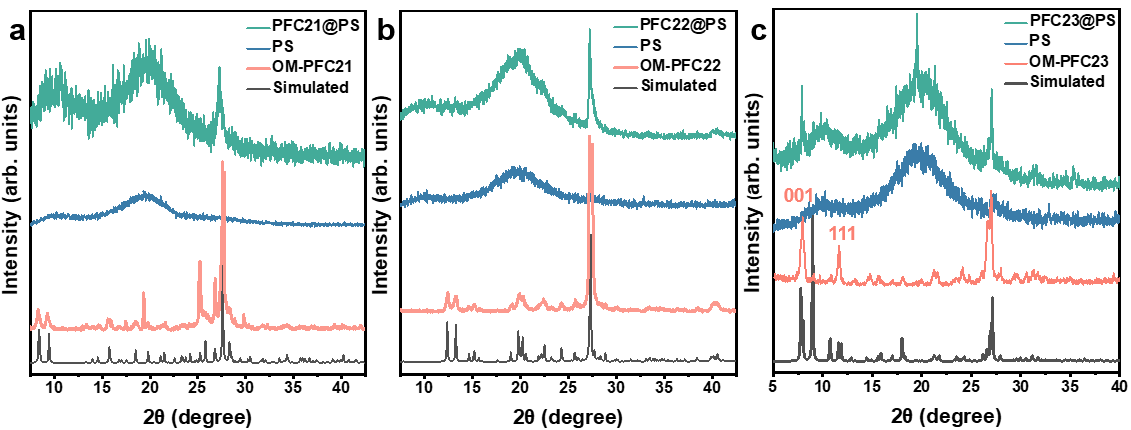
**

**Supplementary Figure 3** **| Structure of** **the** **samples.** The PXRD patterns of (a) PS, PFC21@PS, OM-PFC21 and simulated PFC21, (b) PS, PFC22@PS, OM-PFC22 and simulated PFC22 and (c) PS, PFC23@PS, OM-PFC23 and simulated PFC23.

1. **N_2_ adsorption-desorption isotherms**

**
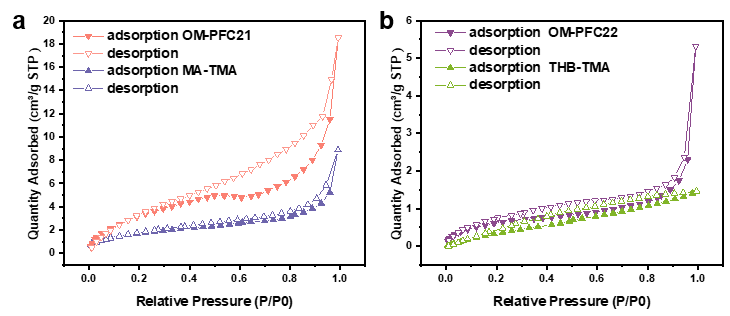
**

**Supplementary Figure 4 |** **N_2_ adsorption-desorption isotherms.** The N_2_ adsorption-desorption isotherms measured at 77 K (a) OM-PFC21 and MA-TMA co-crystal, (b) OM-PFC22 and THB-TMA co-crystal. The BET surface area is ~13 m^2^/g for OM-PFC21, ~6 m^2^/g for MA-TMA co-crystal, ~2 m^2^/g for OM-PFC22, ~1 m^2^/g for THB-TMA co-crystal.


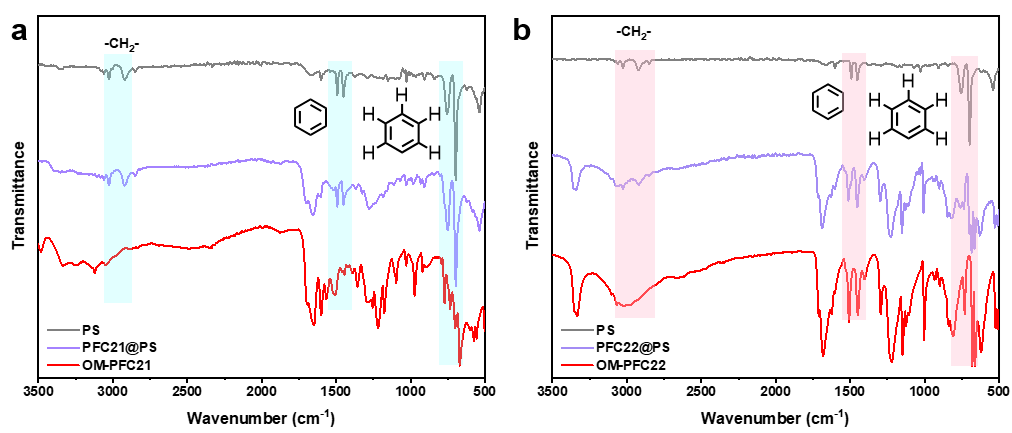


**Supplementary Figure 5** **| Fourier transform infrared (FT-IR) spectra.** The FT-IR Spectra of (a) OM-PFC21, PS and PFC21@PS, (b) OM-PFC22, PS and PFC22@PS. Characteristic peak of the PS template was not observed in spectrum of OM-PFC21 and 22, indicating the complete template removal.


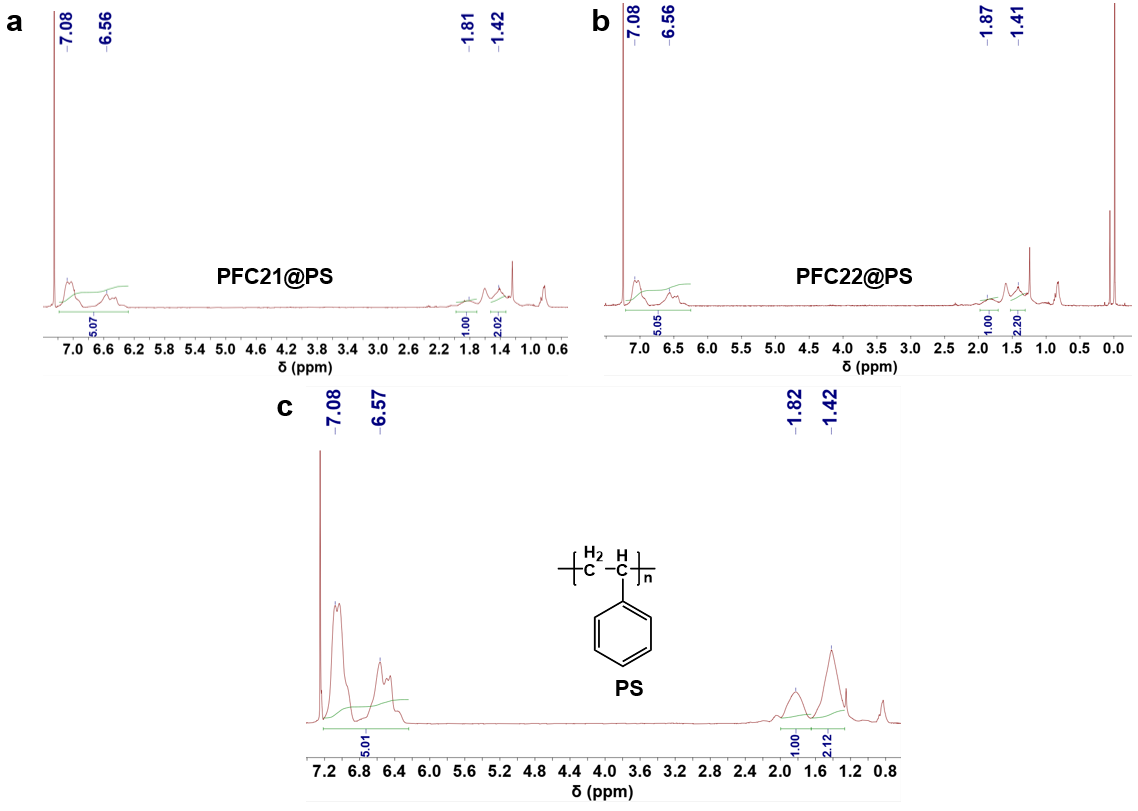


**Supplementary Figure 6 |** **^1^H Nuclear Magnetic Resonance (^1^H NMR) Spectra.** ^1^H NMR spectra of (a) PFC21@PS, (b) PFC22@PS and (c) PS. These composites were soaked in deuterated chloroform for test after the insoluble solids being filtered out.

**
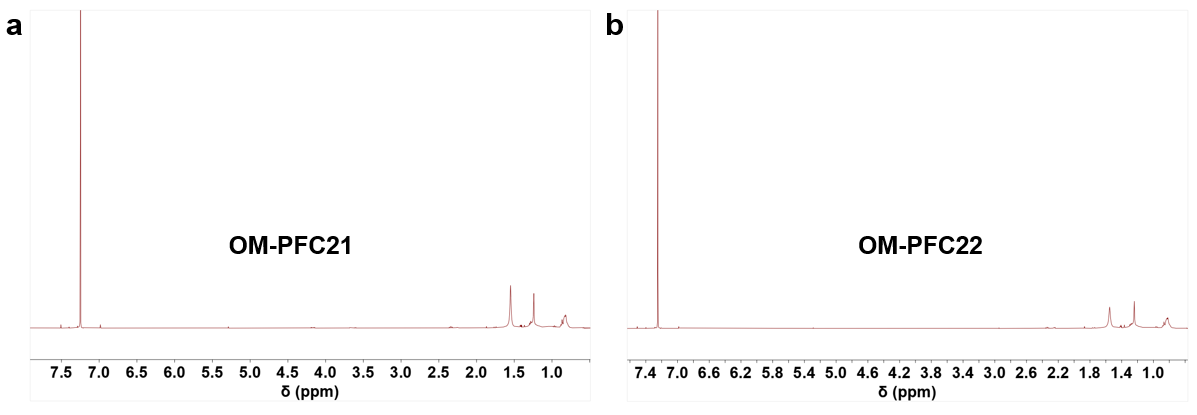
**

**Supplementary Figure 7** **| ^1^H Nuclear Magnetic Resonance (^1^H NMR) Spectra.** ^1^H NMR spectra of (a) OM-PFC21 and (b) OM-PFC22. OM-PFC was immersed in deuterated chloroform solution. No peaks derived from polystyrene were found, indicating the complete template removal.


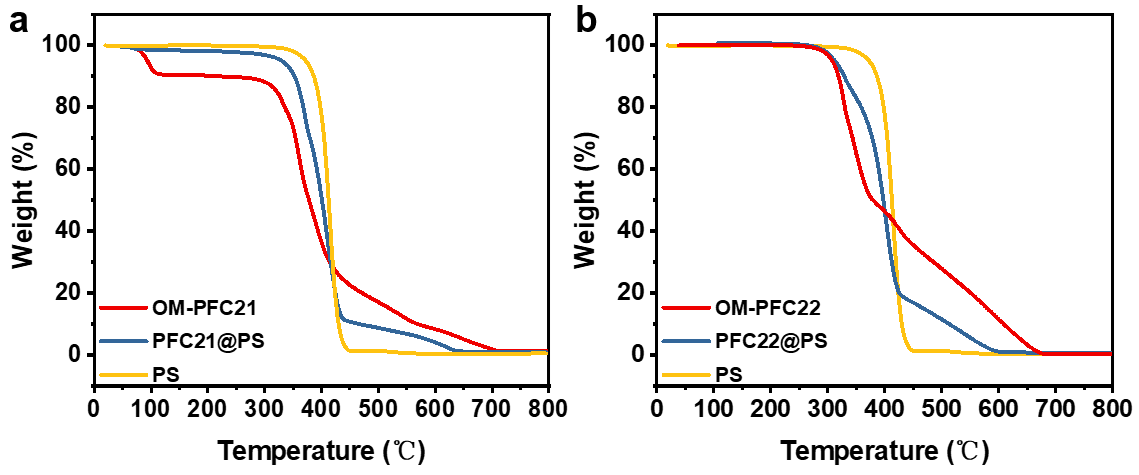


**Supplementary Figure 8 | Thermo-gravimetric Analysis (****TGA) curves.** TGA curves of OM-PFC21, PS and PFC21@PS, (b) OM-PFC22, PS and PFC22@PS.

1. **Scanning electron microscopy (SEM) and transmission electron microscopy (TEM)**


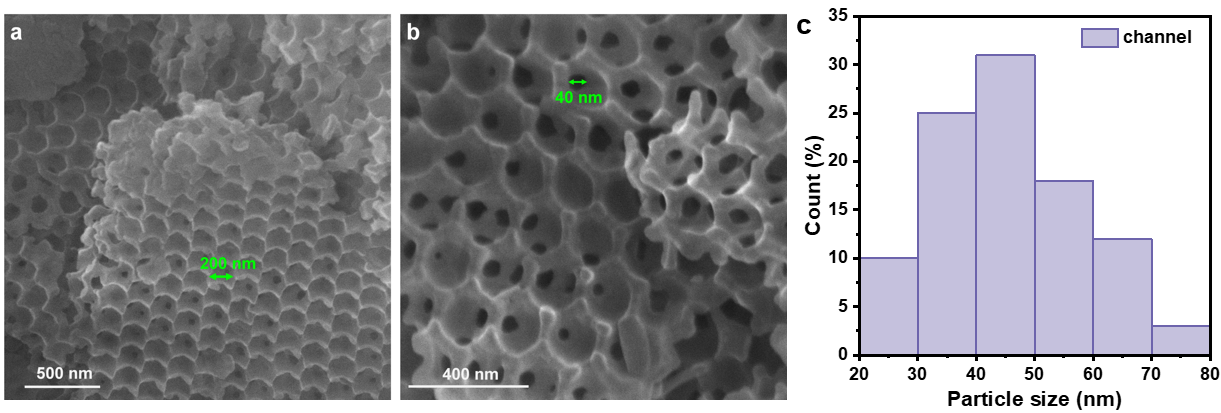


**Supplementary Figure 9** **| Scanning electron microscopy (SEM).** SEM images of OM-PFC. The well-arranged spherical macropores with size about 200 nm were interconnected by around 40 nm channel (based on the average diameter of 100 channels selected at different locations. 3 independent experiments were repeated with similar results).

**
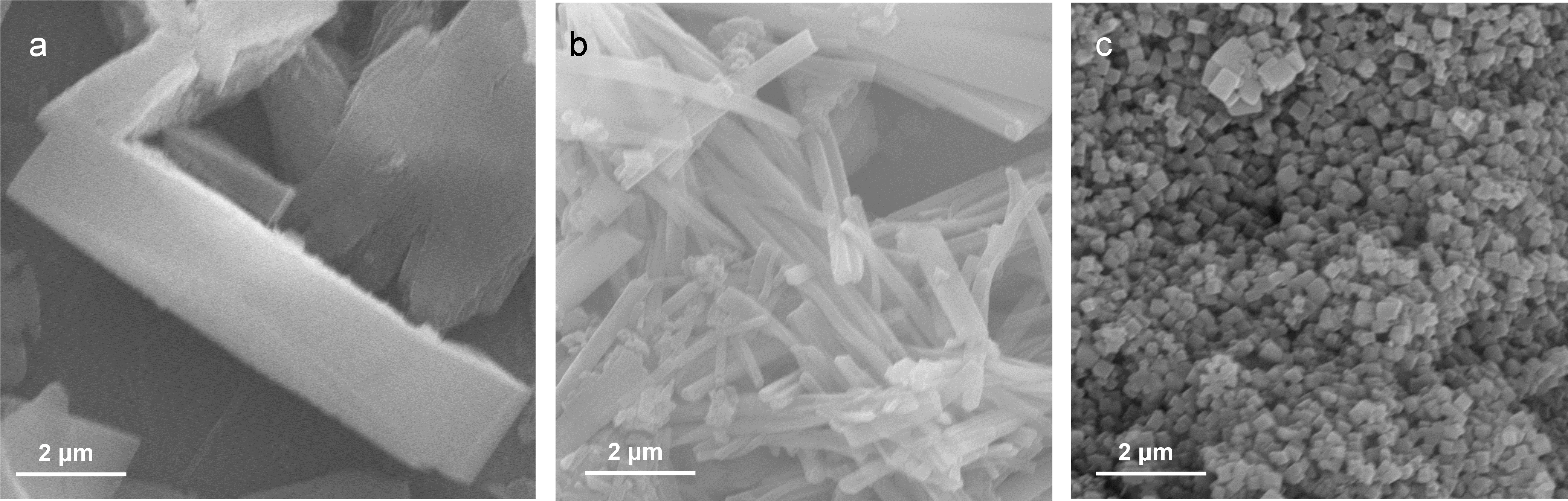
**

**Supplementary Figure 10** **| Morphology of the samples.** SEM images of (a) MA-TMA, (b) THB-TMA and (c) MBU co-crystal (3 independent experiments were repeated with similar results). Scale bar: 2 μm.

**
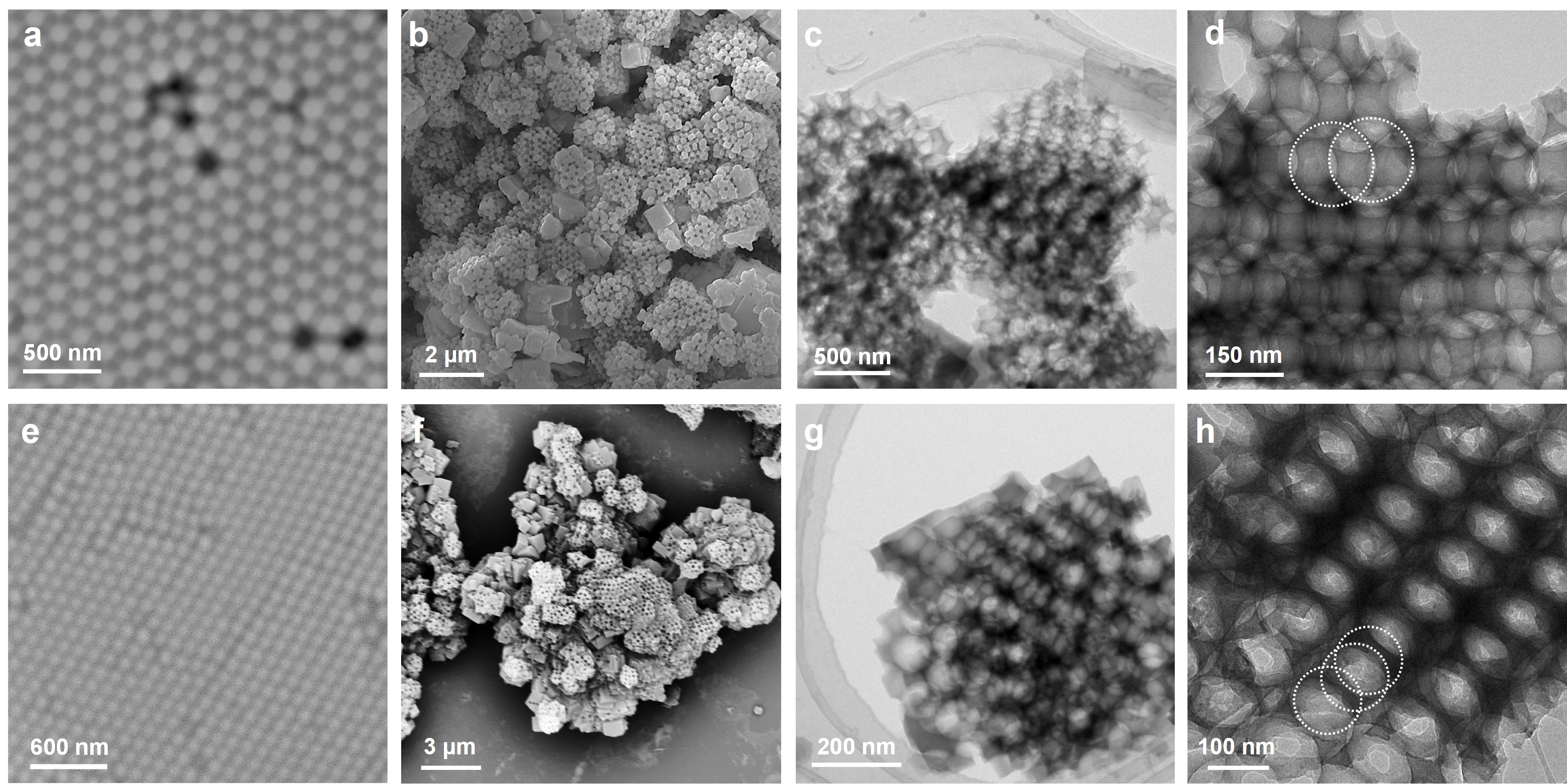
**

**Supplementary Figure 11** **| Morphology of the samples.** The SEM images of (a) PS monolith (Scale bar: 500 nm), (b) OM-PFC23 (Scale bar: 2 μm), TEM images of (c-d) OM-PFC23 synthesized using 150 nm diameters PS (Scale bar: 150 nm). The SEM images of (e) PS monolith (Scale bar: 600 nm), (f) OM-PFC23 (Scale bar: 3 μm), TEM images of (g-h) OM-PFC23 synthesized using 100 nm diameters PS (Scale bar: 200 nm,100 nm). 3 independent experiments were repeated with similar results.


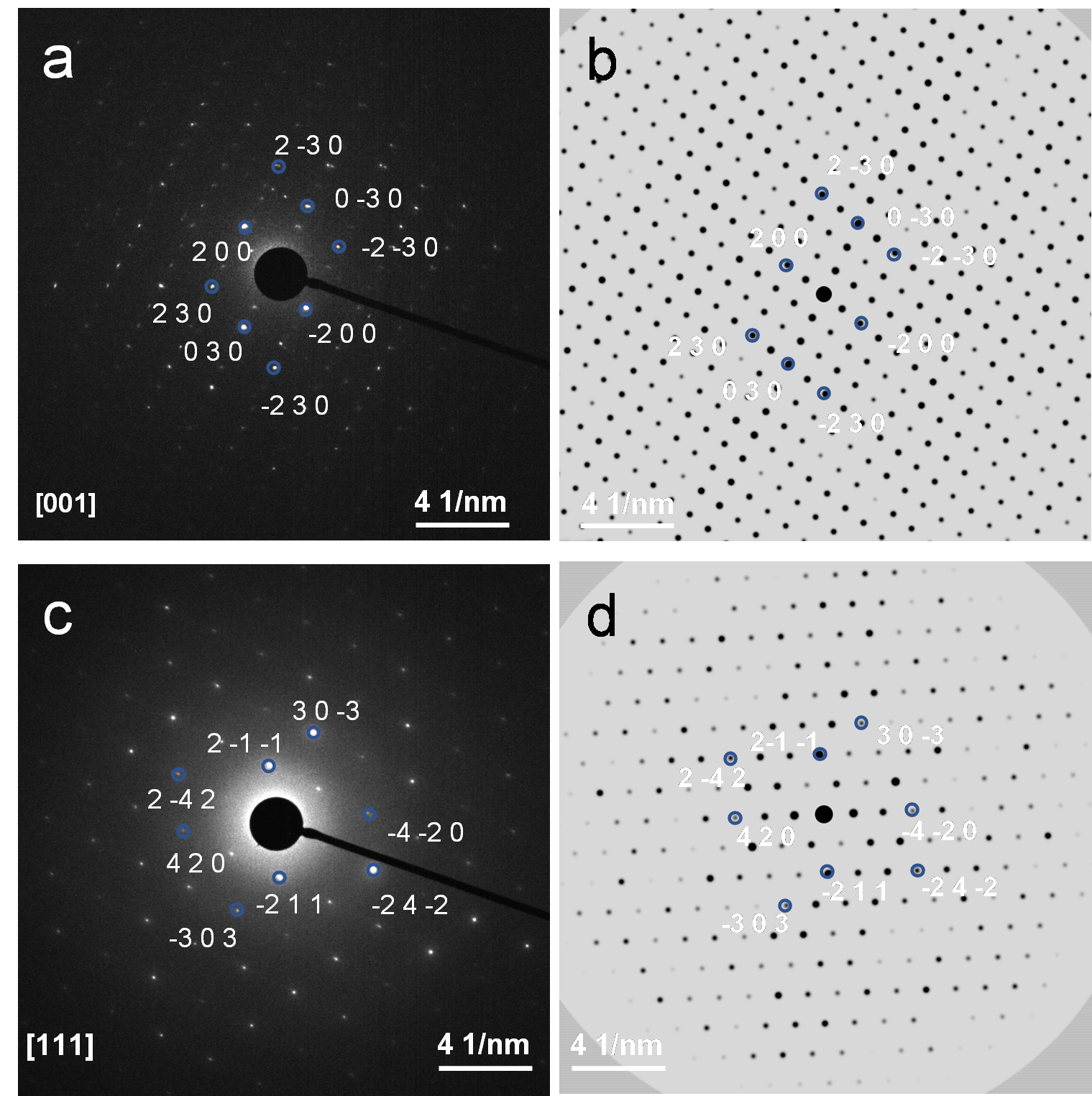


**Supplementary Figure 12 | Selected-area electron diffraction (SAED) patterns.** SAED patterns recorded along [001] plane for (a) OM-PFC23 and (b) the predicted lattice planes based on CIF of MBU crystals^1^. (c, d) show the observed and predicted lattice planes recorded along [111] direction, respectively. (Scale bar: 4 1/nm) The alignment of the observed lattice planes with predicted ones based on MBU single crystal x-ray diffraction confirm the identical crystal structures of OM-PFC23 and MBU.

1. **Solvent stability**

**
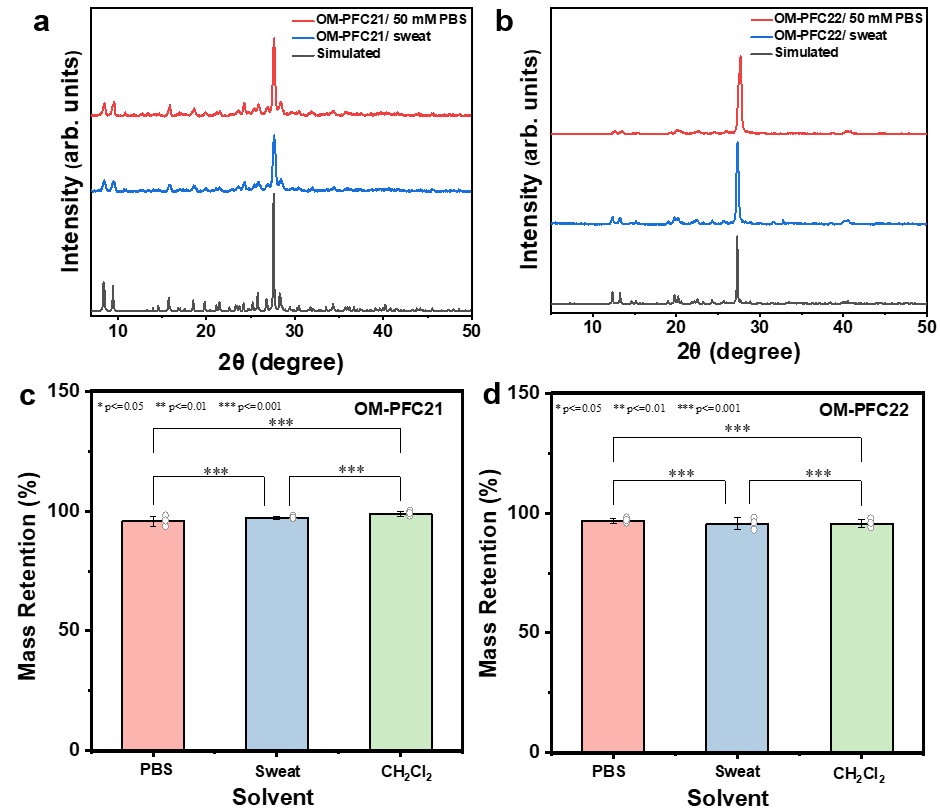
**

**Supplementary Figure 13 | Structural stability.** The PXRD patterns of (a) OM-PFC21 and (b) OM-PFC22 soaked in 50 mM PBS and artificial sweat for a week. The mass retention ratio of (c) the OM-PFC21 and (d) OM-PFC22 before and after soaking in 50 mM PBS, artificial sweat and CH_2_Cl_2_ for a week (n = 3 independent experiments, data are presented in the form of mean values ± SD, * p<=0.05; ** p<=0.01; *** p<=0.001).


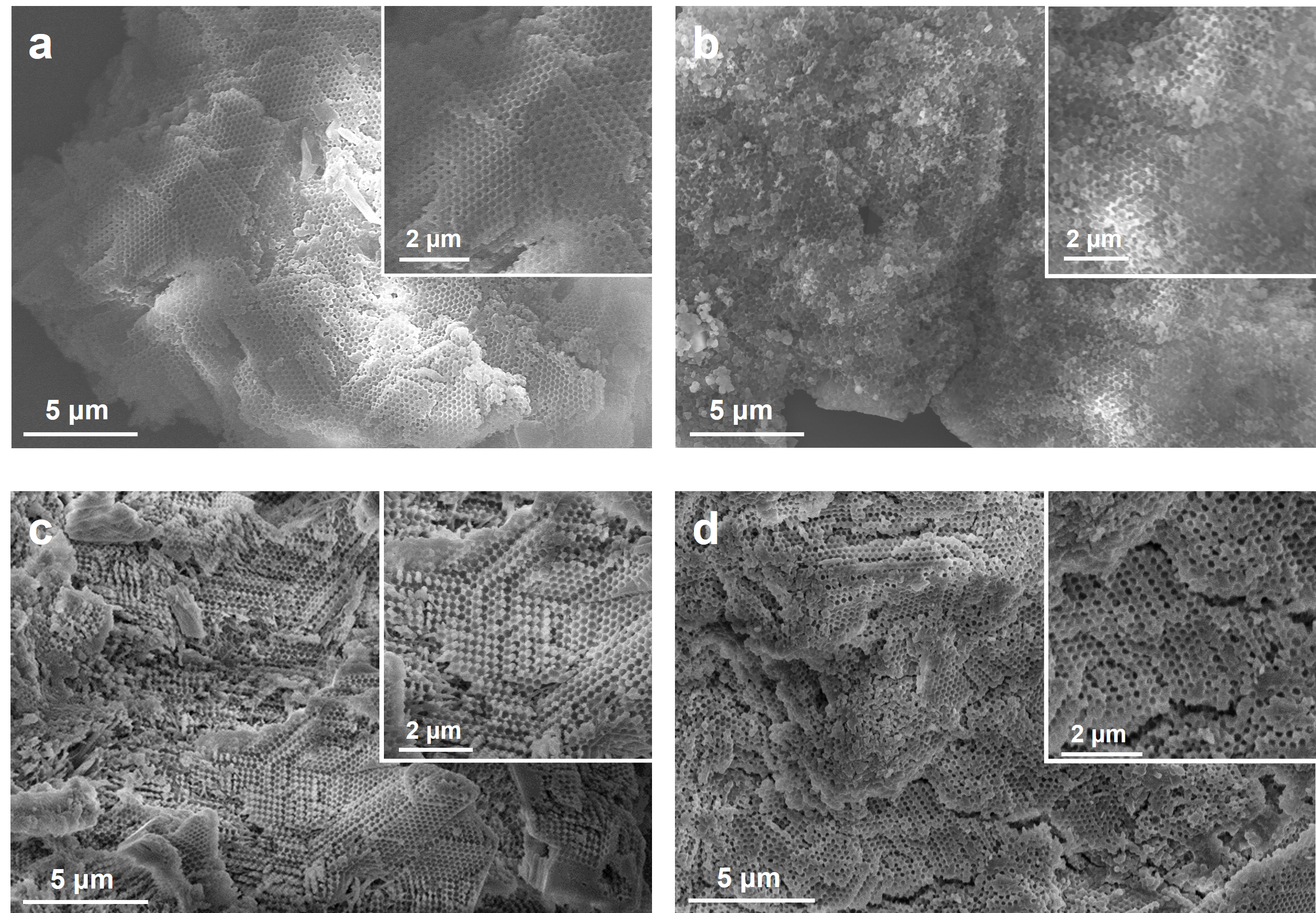


**Supplementary Figure 14 | Morphological stability.** SEM images of (a-b) OM-PFC21 and (c-d) OM-PFC22 soaked in 50 mM PBS and artificial sweat for a week (Scale bar: 5 μm, insert 2 μm). 3 independent experiments were repeated with similar results.

**
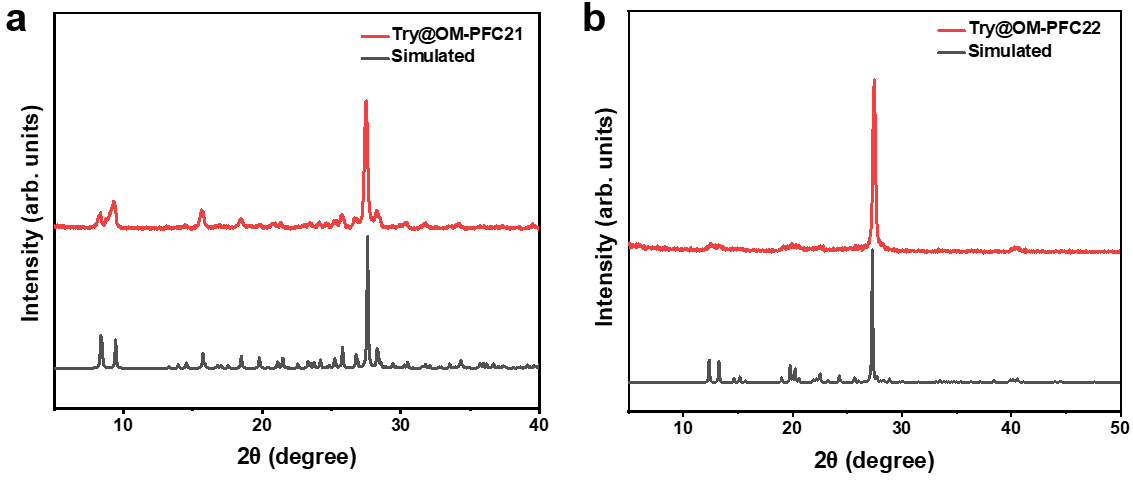
**

**Supplementary Figure 15 | Structure of the samples.** The PXRD patterns of (a) Try@OM-PFC21 and (b) Try@OM-PFC22.

1. **Fourier transform infrared (****FT-IR) spectra**


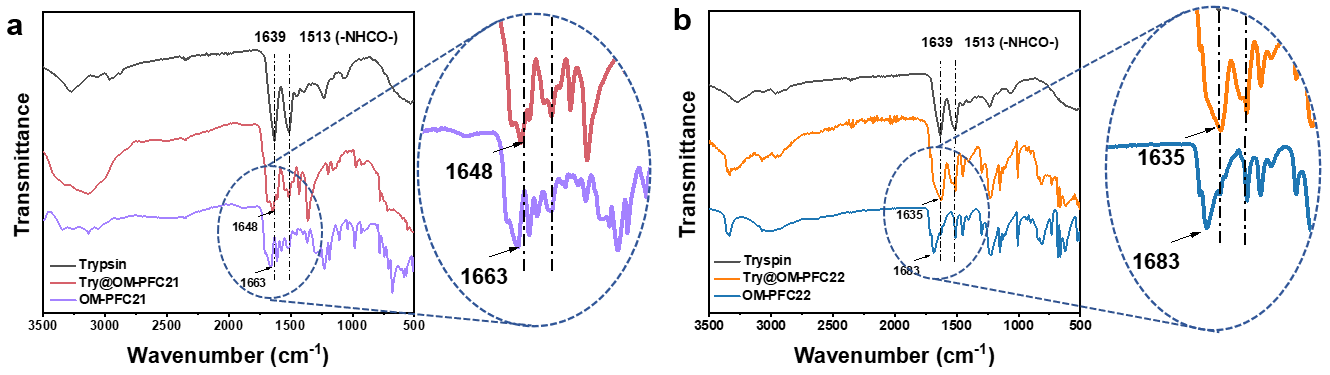


**Supplementary Figure 16 | Fourier transform infrared (FT-IR) spectra.** The FT-IR Spectra of (a) OM-PFC21, Trypsin and Try@OM-PFC21; (b) OM-PFC22, Trypsin and Try@OM-PFC22.

1. **Ultraviolet-Visible (UV-Vis) spectroscopy**

**Trypsin amount determination**

First, a calibration curve correlating absorbance with trypsin concentration was established following the Beer-Lambert law to construct a standard equation. Then the absorbance of trypsin in the solution after immobilization was measured using UV-vis spectroscopy. The residual trypsin content in the solution was then calculated based on the standard equation. Finally, the amount of immobilized trypsin (*Q*) was calculated using equation (1).

*Q* = (*c_0_* −*c_t_*) *V/m* (1)

Where *c_0_* and *c_t_* represent the initial trypsin concentration (10, 20, 40, 60, and 80 mg·mL^-1^) and the residual trypsin concentration after time *t*, respectively, *V* is the volume of liquid phase (1 mL), and *m* is the mass of OM-PFC (10 mg).

**Supplementary Figure 17 | Absorbance standard curve.** The calibration curve for trypsin in the 50 mM PBS.


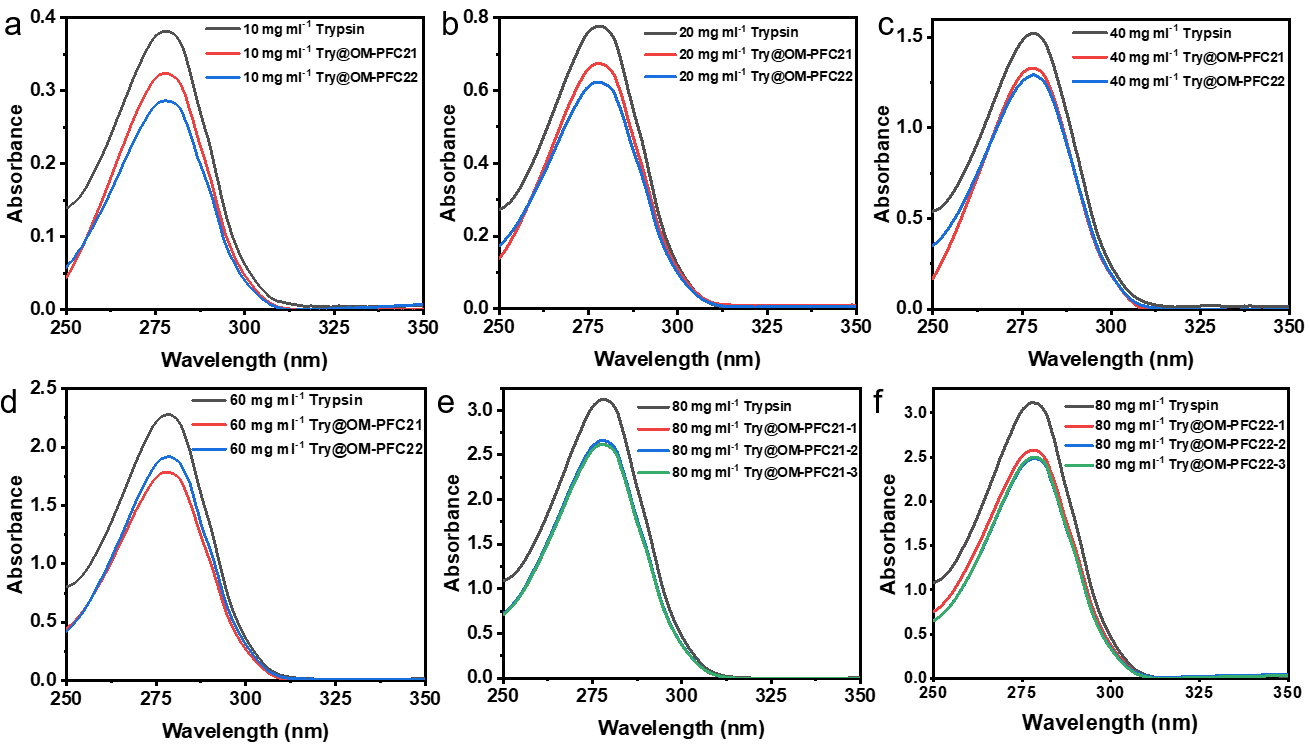


**Supplementary Figure 18 | Loading quantification.** UV-vis absorbance curve for (a-f) OM-PFC21 and OM-PFC22 at different concentrations of trypsin (40-fold diluted prior to test).

**Supplementary Table 2** **| Loading quantification.** The trypsin loading amount on the OM-PFC in a trypsin solution of 80 mg·mL^-1^ (n = 3 independent experiments).

| No. | Try@OM-PFC21  (mg/mg) | Try@OM-PFC22  (mg/mg) |
| --- | --- | --- |
| 1 | **1.14** | **1.54** |
| 2 | **1.36** | **1.36** |
| 3 | **1.26** | **1.5** |
| Average | **1.21** | **1.47** |

**
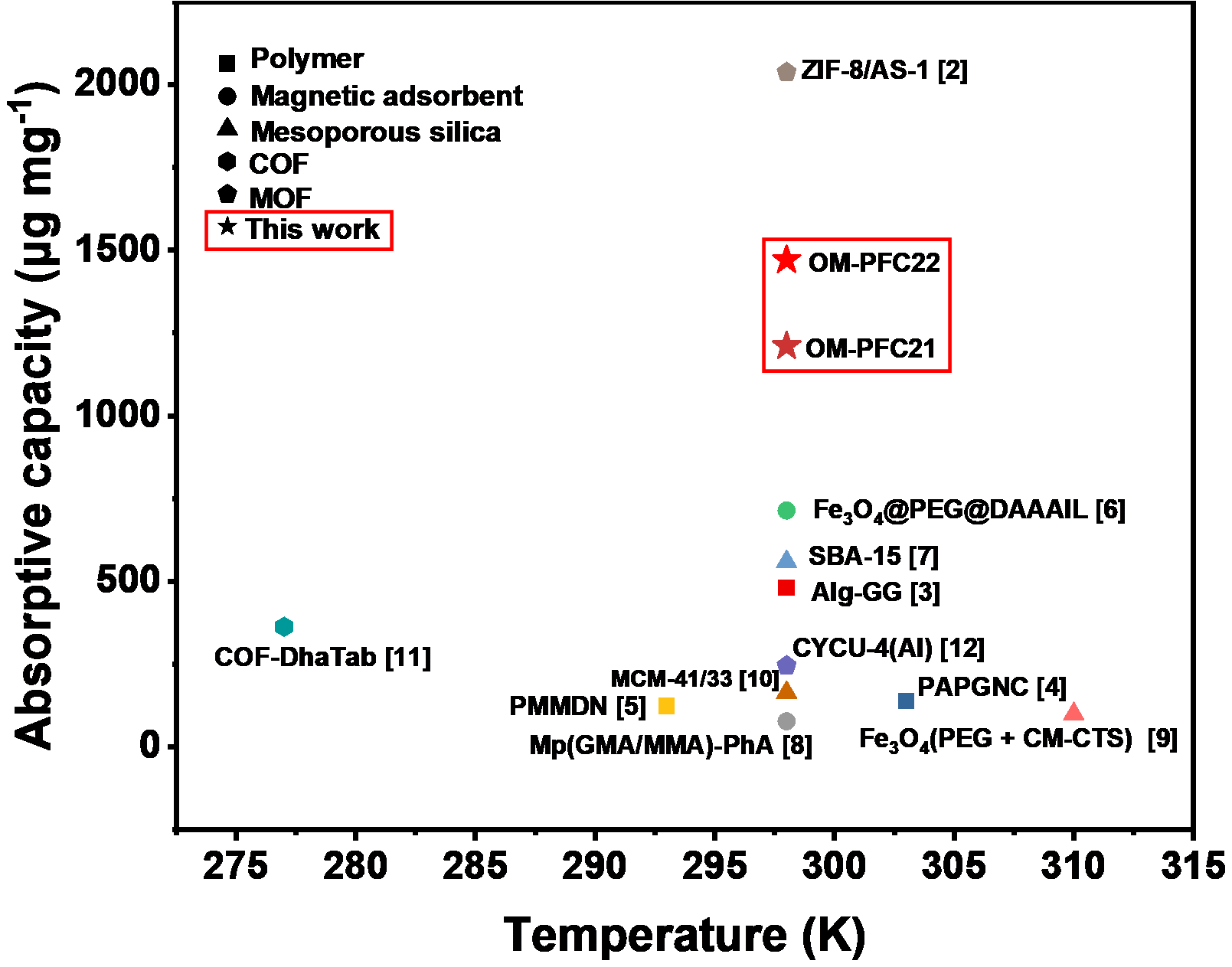
**

**Supplementary Figure 19 | Comparison in adsorption capacity.** The comparison of trypsin adsorption capacity of different materials.^2-12^

**Evaluation of enzymatic activity of free and** **immobilized trypsin**

The activity of free and immobilized trypsin was determined by the hydrolysis rate of the substrate Benzoyl-Arg *p*-nitroanilide (BAPNA), according to the method reported in the literature.^13^ The reaction was carried out in a 50 mM PBS (pH 7.0) at 25 ℃. Briefly, 3 mL of the BAPNA solution (dissolved in DMSO and PBS, v/v 1: 60) was added to a centrifuge tube. The reaction mixture was incubated at 25 ℃, and the assay was initiated by adding the PBS solution of free (20 μL, 10 mg·mL^-1^) or immobilized trypsin (Try@OM-PFC22: 17 μL, 10 mg·mL^-1^, Try@OM-PFC23: 14 μL, 10 mg·mL^-1^). The absorbance of supernatant was measured using a UV-vis spectroscopy at around 380 nm (Supplementary Figure 14). The activity of the trypsin was calculated based on micromoles of paranitroaniline (PNA) formed per gram of trypsin per second.


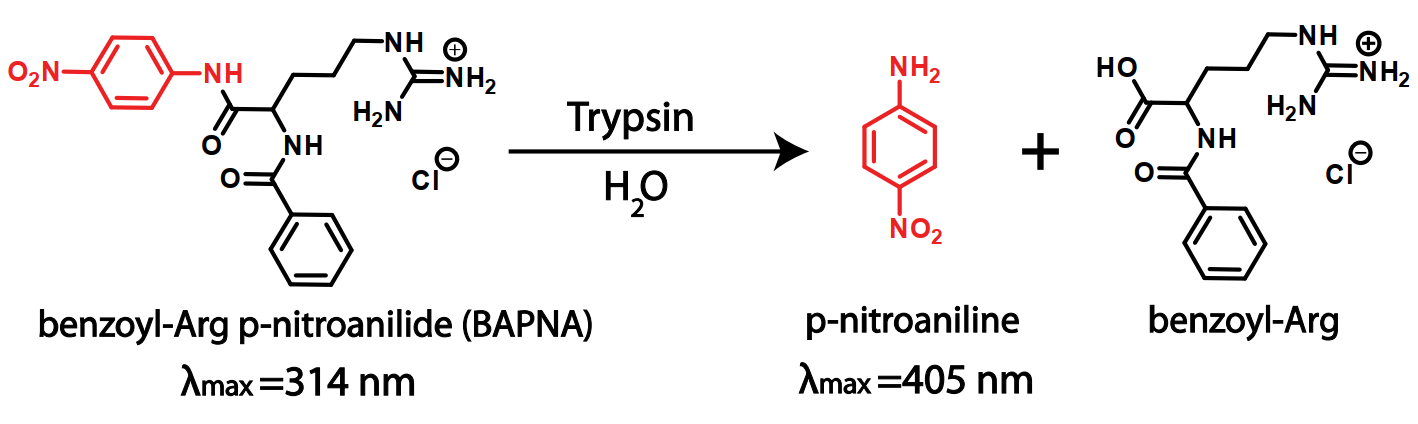

**Supplementary Figure 20 | Absorbance standard curve.** The calibration curve of PNA in 50 mM PBS.

**Michaelis-Menten constants of free and immobilized trypsin**

Michaelis-Menten constants (K_m_) and maximum hydrolysis reaction rates (V_max_) were calculated using the Lineweaver-Burk equation (Eq. (2)) with various concentrations of BAPNA as the substrates.

$V=\frac{V_{max}[S]}{K_{m}+[S]}$ (2)

where [S] is the substrate concentration, V is the hydrolysis reaction rate, K_m_ is Michaelis constant, and V_max_ is the maximum hydrolysis reaction rate.

**Supplementary Figure 21 | Kinetics experiment of** **free trypsin.** (a) The time-course experiment of hydrolysis product (PNA) by trypsin as a function of BAPNA concentration. (b) The Michaelis-Menten plot of free trypsin based on the initial velocity (derived from figure a) versus different concentration of BAPNA solution (trypsin content: 0.2 mg).


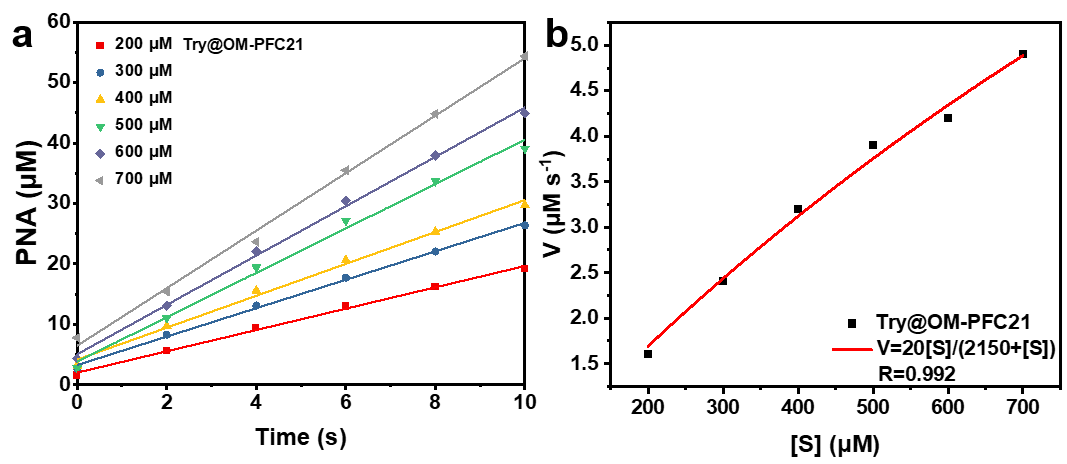


**Supplementary Figure** **22 | Kinetics experiment of Try@OM-PFC21.** (a) The time-course experiment of hydrolysis product (PNA) by Try@OM-PFC21 as a function of BAPNA concentration. (b) The Michaelis-Menten plot of Try@OM-PFC21 based on the initial velocity (derived from figure a) versus different concentration of BAPNA solution (trypsin content: 0.2 mg).


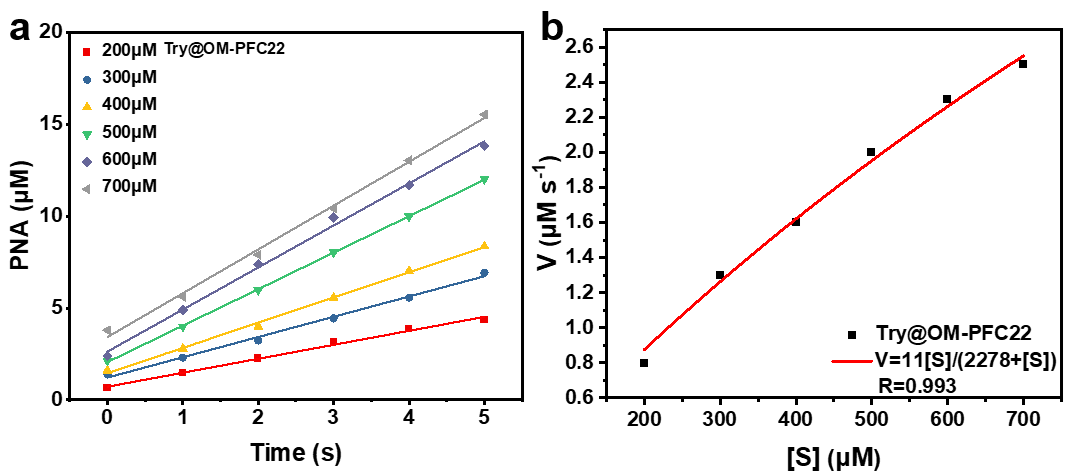


**Supplementary Figure 23 | Kinetics experiment of Try@OM-PFC22.** (a) The time-course experiment of hydrolysis product (PNA) by Try@OM-PFC22 as a function of BAPNA concentration. (b) The Michaelis-Menten plot of Try@OM-PFC22 based on the initial velocity (derived from figure a) versus different concentration of BAPNA solution (trypsin content: 0.2 mg).

1. **Storage stability**

**
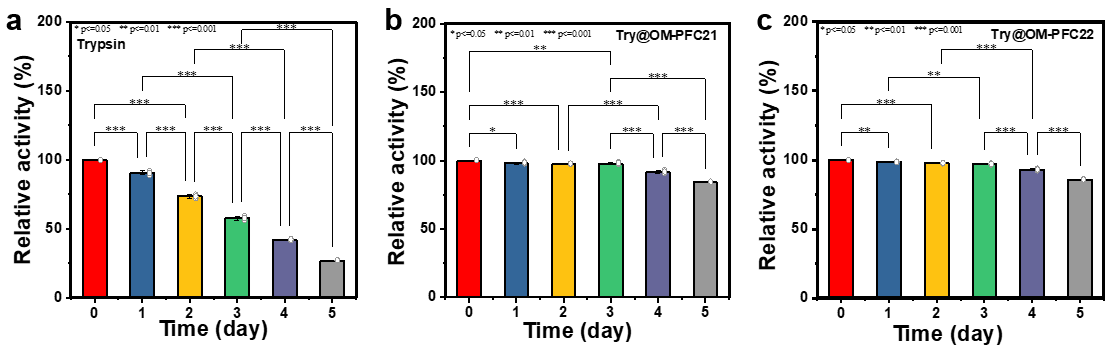
**

**Supplementary Figure 24 | Storage stability.** The storage stability of Try@OM-PFC21, Try@OM-PFC22 and free trypsin (n = 3 independent experiments, data are presented in the form of mean values ± SD, * p<=0.05; ** p<=0.01; *** p<=0.001).

1. **Reusability**

**
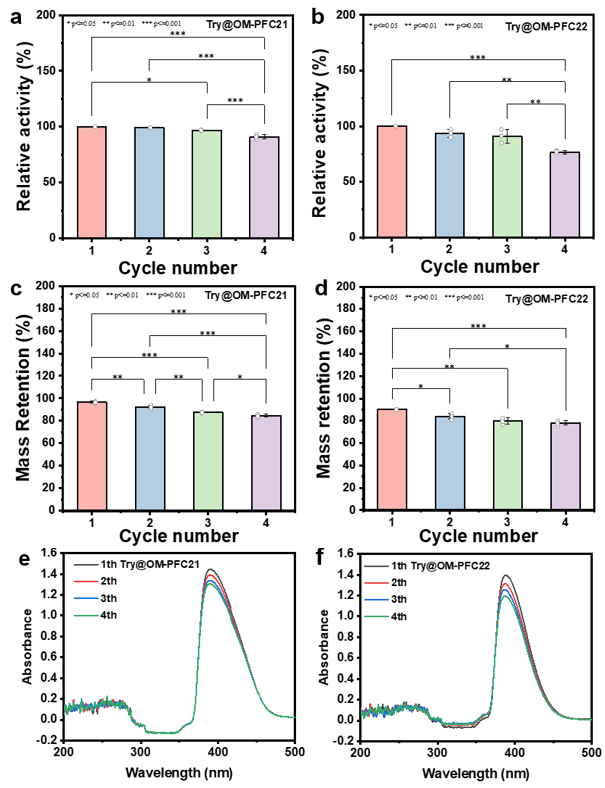
**

**Supplementary Figure 25 | Reusability.** The catalytic activity of recycled (a) Try@OM-PFC21 and (b) Try@OM-PFC22 (n = 3 independent experiments, data are presented in the form of mean values ± SD, * p<=0.05; ** p<=0.01; *** p<=0.001). The mass retention of recycled (c) Try@OM-PFC21 and (d) Try@OM-PFC22 (n = 3 independent experiments, data are presented in the form of mean values ± SD, * p<=0.05; ** p<=0.01; *** p<=0.001), and the UV-vis of (e) Try@OM-PFC21 and (f) Try@OM-PFC22 supernatant. The results indicated a 10~20% weight loss after four recycles caused by the centrifugation operation and resulted in a slight decrease in catalytic activity. UV-Vis spectra of the supernatant showed no significant absorption peak at 280 nm, the characteristic absorption of Trypsin, confirming no enzyme leaching. The above results demonstrate the recyclability of Try@OM-PFC.

1. **Cell culture conditions and fibrocyte differentiation assay**

The Rat PBMCs were washed 2 times with PBS and centrifuged at 1500 rpm for 3 minutes, and the cell pellet was collected. Subsequently, 5 mL of fresh complete medium was added, and the cells were gently resuspended and dispersed. The rat PBMC were cultured in flat-bottomed 96 well tissue culture plates in 250 μL volumes at 2.5×10^5^ cells per mL in a humidified incubator containing 5 % CO_2_ at 37 °C, Try@OM-PFC21 or Try@OM-PFC22 was added to the culture at a final concentration of 12.5 μg·mL^-1^.^14^


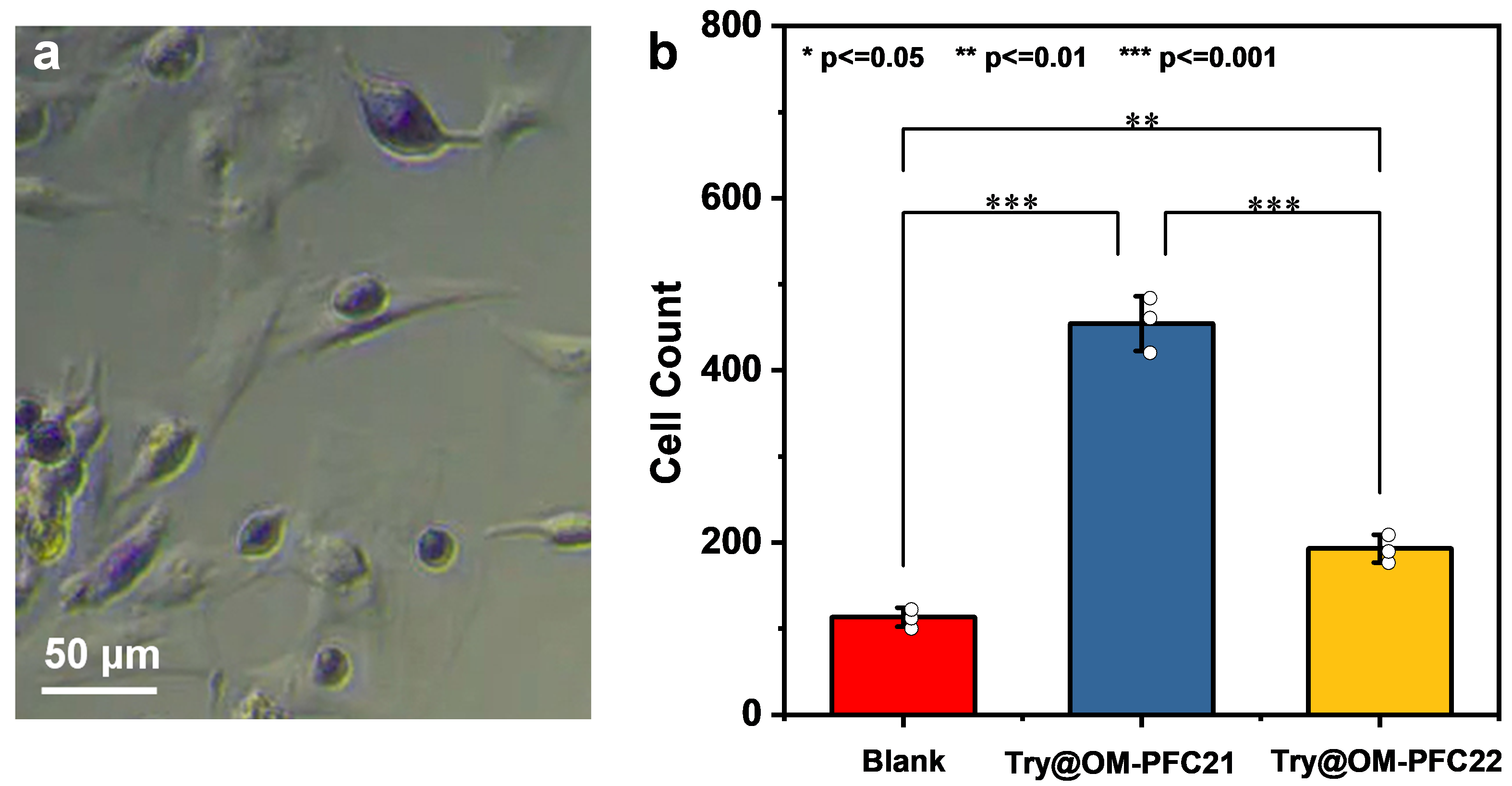


**Supplementary Figure 26 | Morphology and quantification analysis of cell. (a)** After a nine-day incubation of PBMC, the cells were stained with Wright stain. The nucleus was stained blue, fibrous and fusiform shape can be seen clearly. Scale bar: 50 μm. (b) In the original Figure 4a-c, the number of cells was determined by counting in three random fields of view (0.01 mm^2^ per field). n = 3 independent experiments, data are presented in the form of mean values ± SD, * p<=0.05; ** p<=0.01; *** p<=0.001.


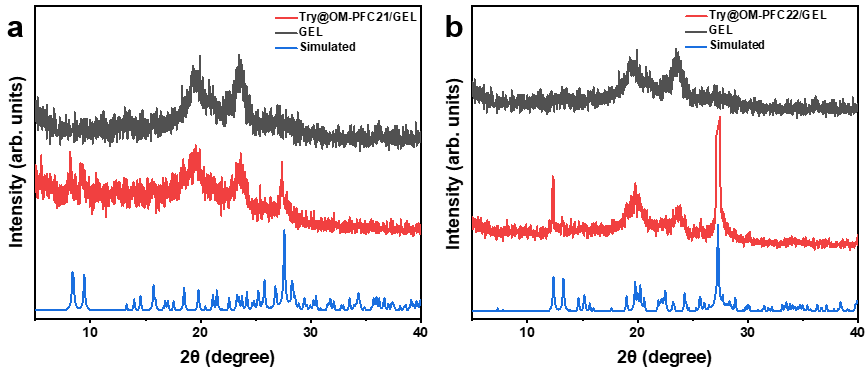


**Supplementary Figure 27 | Structure of the samples.** The PXRD patterns of (a) GEL, Try@OM-PFC21/GEL and simulated PFC21, and (b) GEL, Try@OM-PFC22/GEL and simulated PFC22 after freeze-drying. Try@OM-PFC still remains crystalline in the gel.


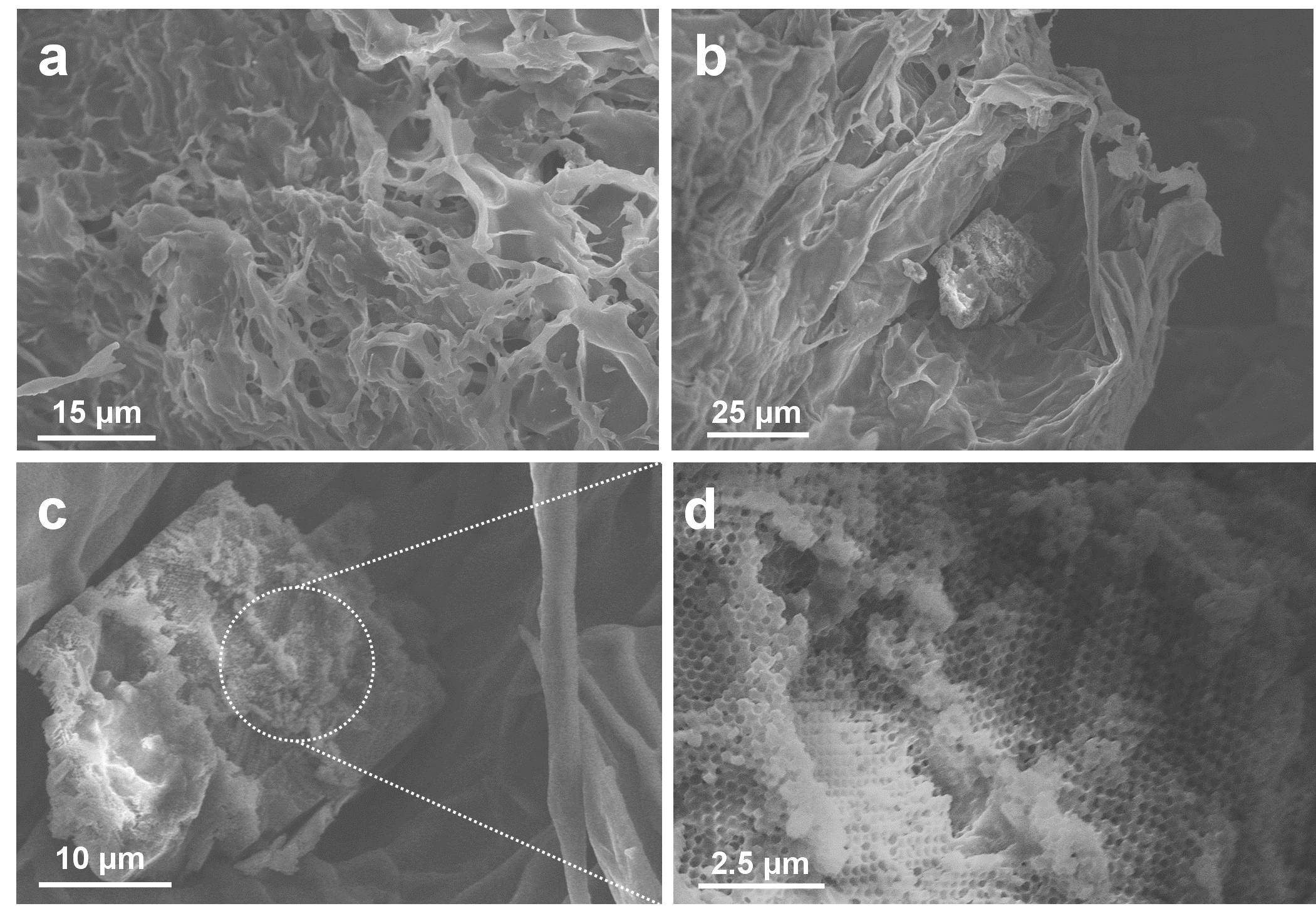


**Supplementary Figure 28** **| Morphology of** **Try@OM-PFC/GEL.** SEM images of Try@OM-PFC/GEL after freeze-drying. Scale bar: (a) 15 μm, (b) 25 μm, (c) 10 μm, (d) 2.5 μm. 3 independent experiments were repeated with similar results.

**Supplementary Figure 29 | Hydrolysis process of Try@OM-PFC/GEL.** (a) The photographs of Try@OM-PFC/GEL dressing immersed in BAPNA solution, showing colorless mixture at the beginning. (b) BAPNA was hydrolyzed to PNA by Try@OM-PFC/GEL dressing with the solution gradually turn to yellow, demonstrating that Try@OM-PFC retained its activity after being incorporated into the flexible matrix. 3 independent experiments were repeated with similar results.


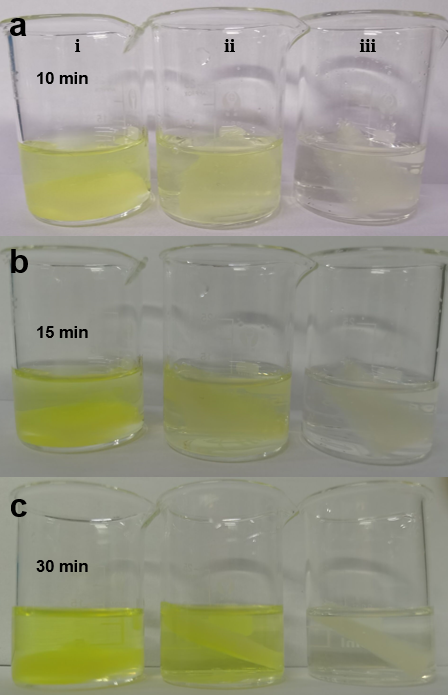


**Supplementary Figure 30 | Comparison of hydrolysis process.** Time-dependent hydrolysis of BAPNA by (i) Try/GEL, (ii) Try@OM-PFC /GEL, and (iii) OM-PFC/GEL control dressings at (a) 10, (b) 15, and (c) 30 min incubation intervals. The above results show that the Try/GEL system demonstrates faster BAPNA-to-PNA hydrolysis rate due to the easily access to catalytic active sites of free trypsin. OM-PFC/GEL without trypsin (negative control) shows no hydrolytic activity, confirming that the catalytic activity is derived from the enzyme. 3 independent experiments were repeated with similar results.

**Supplementary References**

1 Sours, R. E., Fink, D. A. & Swift, J. A. Dyeing uric acid crystals with methylene blue. *J. Am. Chem. Soc.* **124**, 8630-8636 (2002).

2 Lu, J. *et al.* Fabrication of Microporous Metal-Organic Frameworks in Uninterrupted Mesoporous Tunnels: Hierarchical Structure for Efficient Trypsin Immobilization and Stabilization. *Angew. Chem. Int. Ed.* **59**, 6428-6434 (2020).

3 Aravena, P., Emilia Brassesco, M., Bosio, B., Pico, G. & Woitovich Valetti, N. Chemically Modified Alginate Bead Matrix for Efficient Adsorptive Recovery of Trypsin from Fresh Bovine Pancreas. *Biotechnol. Progr* **34**, 1269-1277 (2018).

4 Anirudhan, T. S. & Rejeena, S. R. Adsorption and hydrolytic activity of trypsin on a carboxylate-functionalized cation exchanger prepared from nanocellulose. *Colloid Interface Sci* **381**, 125-136 (2012).

5 Wang, H., Wan, J. & Cao, X. Preparation of a pH-sensitive affinity precipitation polymer and its application in purification of trypsin. *Sep. Purif. Technol* **68**, 172-177 (2009).

6 Yang, Q. *et al.* A novel dianionic amino acid ionic liquid-coated PEG 4000 modified Fe_3_O_4_ nanocomposite for the magnetic solid-phase extraction of trypsin. *Talanta* **174**, 139-147 (2017).

7 Karimi, B., Emadi, S., Safari, A. A. & Kermanian, M. Immobilization, stability and enzymatic activity of albumin and trypsin adsorbed onto nanostructured mesoporous SBA-15 with compatible pore sizes. *Rsc Advances* **4**, 4387-4394 (2014).

8 Bayramoglu, G., Ozalp, V. C. & Arica, M. Y. Magnetic Polymeric Beads Functionalized with Different Mixed-Mode Ligands for Reversible Immobilization of Trypsin. *Ind. Eng. Chem. Res* **53**, 132-140 (2014).

9 Sun, J., Hu, K., Liu, Y., Pan, Y. & Yang, Y. Novel superparamagnetic sanoparticles for trypsin immobilization and the application for efficient proteolysis. *J Chromatogr B* **942**, 9-14 (2013).

10 Goradia, D., Cooney, J., Hodnett, B. K. & Magner, E. The adsorption characteristics, activity and stability of trypsin onto mesoporous silicates. *Mol. Catal. B Enzym* **32**, 231-239 (2005).

11 Kandambeth, S. *et al.* Self-templated chemically stable hollow spherical covalent organic framework. *Nat. Commun.* **6** (2015).

12 Liu, W.-L. *et al.* Fast Multipoint Immobilized MOF Bioreactor. *Chem. Eur. J* **20**, 8923-8928 (2014).

13 Kandambeth, S. *et al.* Self-templated chemically stable hollow spherical covalent organic framework. *Nat. Commun.* **6**, 6786 (2015).

14 Pilling, D., Vakil, V. & Gomer, R. H. Improved serum-free culture conditions for the differentiation of human and murine fibrocytes. *J. Immunol. Methods* **351**, 62-70 (2009).
